# The time is now: why we must identify and address health disparities in sport and recreation injury

**DOI:** 10.1186/s40621-021-00320-2

**Published:** 2021-06-14

**Authors:** Charlotte Baker, Oziomachukwu Chinaka, Elizabeth C. Stewart

**Affiliations:** 1 Department of Population Health Sciences, Virginia-Maryland College of Veterinary Medicine, Virginia Tech, Blacksburg, VA USA; 2 Department of Surgery, Meharry Medical College, Nashville, TN USA

**Keywords:** Social determinants of health, Sports injury, Recreation, Methodology, Data collection, Health disparities, Health equity

## Abstract

**Background:**

Social and structural determinants of health (SDOH) are the conditions in which individuals are born, live, learn, work, play, worship, and age. These drivers of health are integral in contextualizing the understanding and prevention of sport and recreation injury (SRI), and recognizing their impact is necessary to provide a complete and accurate picture of health and health outcomes related to injury.

**Main:**

Reducing disparities and achieving equity in sports and recreation is possible in part by improving data collection methodologies and utilization. Often, many SDOH have considerable effect on SRI. Although SRI epidemiology frequently examines differences by sex, there is limited inclusion of factors such as socioeconomic status, housing, gender, and food security, in sport specific data sources or in analysis of sport recreation and injury using other sources (e.g. administrative data). The ongoing dual epidemics in the United States – racism and COVID-19 – have emphasized the importance of having and utilizing SDOH data to reduce the burden of injury and disproportionate effects on our diverse population.

**Conclusion:**

Moving forward, to address disparities in SRI, SDOH must be included as a part of research priorities, health related goals, and policies. This difference can be made in developing consistency in data collection and utilization. This will provide an accurate picture of the intersections and interdisciplinary changes required to design the best approach to problems to develop solutions. Future data collection and utilization should prioritize SDOH.

## Background

Significant inequities exist in injury prevention and control in the US as demonstrated by health disparities across age, race, ethnicity, region, and sex for motor-vehicle crashes, homicide, suicide, traumatic brain injuries, drug use, and work-related injuries (Daugherty et al. 2019; National Academies of Sciences, Engineering, and Medicine, et al. n.d.; Moore et al. 2019; CDC Health Disparities and Inequalities Report (CHDIR) - Minority Health - CDC n.d.). Health disparities are differences that are “closely linked with social, economic, and/or environmental disadvantage” (Healthy People 2020 Disparities 2020) and “affect groups of people who have systematically experienced greater obstacles to health based on their racial or ethnic group; religion; socioeconomic status; gender; age; mental health; cognitive, sensory, or physical disability; sexual orientation or gender identity; geographic location; or other characteristics historically linked to discrimination or exclusion” (U.S. Department of Health Human Services 2010; U.S. Department of Health and Human Services 2008). It is very important to note that we distinguish sex from gender. Sex is an assignment and classification system used at birth based on “anatomy, hormones, and chromosomes” and the classifications most often include male, female, and intersex (The Gender Unicorn n.d.). Gender (and gender identity) on the other hand, is who a person is - the “internal sense of being male [man/boy], female [woman/girl], neither of these, both, or another gender (s)” and is not necessarily linked to sex (The Gender Unicorn n.d.). Both of these are different than gender expression, physical attraction, and emotional attraction, concepts that are not discussed here.

Sport and recreational injury (SRI) epidemiology frequently examines differences by sex, yet there is limited population level literature (U.S. Department of Health Human Services 2010; Lyons et al. 2019; Wallace et al. 2020a; Wallace et al. 2020b; Waterman et al. 2012) outside of the sociology of sport (DeLuca 2013; Hermann and Vollmeyer 2016; Tate et al. 2015; Slater and Tiggemann 2010; McHale et al. 2005; Wiley et al. 2000; Atkinson and Martin 2020) that demonstrates the regular inclusion of other social and structural determinants of health (SDOH), e.g. gender, disability, food insecurity, race, or ethnicity in data collection or as explanatory factors for SRI despite an emphasis on capturing and using factors such as gender by the US Department of Health and Human Services (Healthy People 2030; Institute of Medicine (US) Board on the Health of Select Populations n.d.; Sex and Gender n.d.). There are many reasons we have disparate outcomes due to SDOH (Trent et al. 2019) – people have long term stressors from SDOH that lead to chronic stress; the body’s reaction to chronic stress increases the risk of chronic disease; where people reside can have a detrimental effect on net wealth, availability of equal opportunities and services that are essential for better jobs and health; and stressors can be passed from one generation to another (Trent et al. 2019; Rexing et al. 2020; Saunders et al. 2016). These reasons are all connected to participation and injury rates. Knowing the impact of SDOH on SRI through research can provide an evidence base necessary to create or change policy to prevent initial injury or re-injury; improve training for clinicians that interact with athletes to prevent initial injury (e.g. athletic trainers, primary care) or improve chances of return after injury (e.g. athletic trainers, orthopedists, physical therapists); reduce disparities in sport training and clinical treatment; and identify precisely where sport can intercede to minimize or eliminate this effect. If we want to prevent injuries, we must alter the root causes of injury. Blanket recommendations are not as effective as targeted recommendations. Because SDOH are critical to understanding and preventing SRI, we must gather appropriate data and address health disparities. For the purpose of this commentary, community sport or community recreation refers to any physical activity done for fun, whether organized or not, e.g. skateboarding or pickleball tournament at a retirement home. We define elite sport (Williams 2017) as those competing at the highest level of their sport (e.g. athletes participating in international competitions).

## Main

### Data collection in sports injury epidemiology

There are many SDOH that factor into the occurrence of SRI. For example, environmental conditions affect the incidence of skiing injuries (Pierpoint et al. 2020) (physical environment), a high school’s size impacts rates of injuries (King et al. 2015) (neighborhood environment), and policies are used to discriminate against transgender athletes in organized sport (Jones et al. 2017) (social integration). We must curate information sources about these determinants as they apply to sport and recreation.

Of the 330.5 million people (U.S. and World Population Clock n.d.) in the US, approximately 40% identify as belonging to a racial or ethnic minority population; more than 13% have a disability; approximately 4% identify as Lesbian, Gay, Bisexual, or Transgender (In U.S., More Adults Identifying as LGBT n.d.); and 9% are uninsured (United States Quick Facts n.d.). More people are now aware of a long existing epidemic of racism in the US and globally as well as health issues that have been linked to it, xenophobia, and class-based policies. Some may argue that SRI are mostly biological or situational in nature and thus “upstream” factors such as SDOH are not significant enough to be collected or used for prevention. The very situation in question is driven by who is able to participate and how. How we medically handle injuries after they occur is driven by what resources are available. A high school basketball player on Medicaid or a parent or guardian’s high deductible health insurance plan and no disposable income who breaks their leg may eventually return to play, but the time to return could be affected by the affordability, distance, and timing of care. A high school player with a parent or guardian with a low deductible private health insurance plan and disposable income however, could have immediate access to the best treatment available despite cost and distance to said treatment.

Data can be used to highlight these types of health disparities and improve health equity. Primary data sources must begin to collect these factors and report on them. Researchers must harness the available SDOH information in explanatory and prediction models. While there is currently limited data for community sport, injuries that result in contact with a clinician are captured in billing data and reported to state and federal agencies. This data typically contains geographic, race, ethnicity, and insurance information, yet few states collect information on gender and it is not always available for research use. Just as with race or economic data, collecting data on gender is important to ensure that we can identify and prevent marginalization of people (Tan et al. 2020), and see where adjustments in the structure of community or elite sport can maintain or increase inclusion and participation (Roberts and Christens 2020; Lett et al. 2020). In sport, sex-testing (Ritchie et al. 2008; The Foundation Position n.d.) particularly of female and intersex athletes is pervasive and based on outdated notions of who someone is and what they are capable of (Pieper 2016; Clifton 2020). Using gender (The Gender Unicorn n.d.) as opposed to or in conjunction with sex (Institute of Medicine (US) Board on the Health of Select Populations n.d.) will help in efforts to improve health access, outcomes, and treatment for everyone (Christian et al. 2018; Gonzales and Henning-Smith 2017). If we do not collect the information that we are missing, it is easy to say that those groups do not exist and thus discrimination and disparities do not exist. Collaboration between academic institutions, states, and federal health agencies could improve the availability of this data.

There are obvious issues with setting up data collection – does an individual get to self-report these factors or is there someone who will “assign” a value to a person? What about data privacy? These are regular issues that data analysts and epidemiologists have to work with. It makes sense to have individuals self-report as much SDOH information as possible (e.g. gender, race, ethnicity, disability, barriers for participation), and researchers validate when it makes logical sense to do so (e.g. income, geography). Data use agreements should always consider protecting the privacy of individuals. Deciding who should and how to collect information about community sport has not been determined. Discrimination has made it difficult to encourage discussion of gender or disability, let alone collection of this information. Given the difference between gender and sex, we must find ways to move beyond this. While several US cities and states have validated questions to capture gender, more work needs to be done (Herman et al. 2017; Patterson et al. 2017). This helps when it comes to collection for the general population, but we must consider the effect of homophobia and transphobia when collecting this information especially in athletic populations. We must also consider the effect of ableism in sport. The inclusion of gender, disability, and other characteristics in data collection is likely to move faster than inclusion in society. Making data collection more common may help move society forward. Collaborations with clinical centers, community organizations, and parent organizations could make this possible.

The utilization of the socioecological model (Bronfenbrenner 1977; Bronfenbrenner 1986) by Runyan (Runyan 1998) and others (Hanson et al. 2005) as well as the Haddon Matrix (Haddon Jr. 1968) gives us room to explore these ideas and be as inclusive as necessary in factors of the individual, their family and friends, their community, and policies that influence participation, injury, and recovery. Despite this, often used data (e.g. NCAA Injury Surveillance Program (NCAA Injury Surveillance Program n.d.); the Nationwide Emergency Department Sample, Healthcare Cost and Utilization Project, Agency for Healthcare Research and Quality (Agency for Healthcare Research and Quality n.d.)) does not include enough information to help with identification of health disparities and decision making for their elimination. Some, such as the Nationwide Emergency Department Sample (Agency for Healthcare Research and Quality n.d.), are compiled from state level data, which in turn are compiled from individual hospital level discharge data. These original sources tend to capture SDOH information creating the possibility of having more data available nationally. Many SRI are seen at an emergency department but the Nationwide Emergency Department Sample in particular does not make SDOH variables such as race or ethnicity available for examination. College athletes that do not fall under the purview of an athletic department (e.g. club sports and intramurals) tend not to be included in surveillance of injury. Collaborations with university recreation and academic departments could make this a true possibility.

Including certain SDOH data (e.g. race, age, insurance) would be easier for the NCAA to require in collection, but other variables such as gender and SES are not easily captured. Things like family history and cultural competency are ripe for qualitative analysis. It can be argued that the college campus setting mitigates or eliminates disparities and thus reduces the need for SDOH collection in NCAA athletics. Being on a college campus does not eliminate food insecurity. Not all sports are treated equally in terms of food access (i.e. training table) and not all colleges have equivalent nutrition training programs for athletes. Non-scholarship athletes do not necessarily have the same financial or food benefits as scholarship athletes. The ability of a family to contribute financially to a student-athlete, whether scholarship or non-scholarship, or their ability to obtain employment while in school can influence the effect of economics on stress and, consequently, injury risk. Health care access includes the ability to afford care, language differences, trust, and cultural competency (Healthy People 2020: Access to Health Services 2020). NCAA athletes must have some form of health insurance whether being included on parent’s insurance (which assumes their parents are eligible and can afford health insurance) or having purchased a plan offered by the athletic department to cover injuries that occur during athletic activity (NCAA Student-Athlete Medical Insurance Legislation n.d.) but this, nor access to a student health center, can be expected to cover all potential costs of care. In terms of race and ethnicity, there is homogeneity in Division I football (NCAA Division I Football Bowl Subdivision 65%) and basketball (NCAA Division I women’s 66%, men’s 75%) (Lapchick 2020). This supports the possibility of statistical interaction by race and ethnicity – different outcomes for different groups. It would be important to look at the outcomes by race or ethnicity rather than providing an overall look at college athletics. Gender is not captured on a wide scale in collegiate athletics and thus we do not know the influence gender and related SDOH have on injury risk or burden.

### Data utilization

The highlighting of the dual epidemics in the US – racism and COVID-19 – brings to light how important it is to a) have SDOH data and b) utilize SDOH data to be sure that not only are certain groups not having a greater burden of injury but also that we are not having disproportionately poor health outcomes due to sport or recreation during this time. If we integrate information on SDOH such as race and gender as explanatory variables with more “traditional” independent variables used in SRI such as playing surface, contact, and insurance status, we can dive deeper into the root causes and intersections of why someone is and is not participating, injured, or recovering and returning to sport. Intersectionality, first used by Kimberlé Crenshaw in 1989 (Crenshaw 1989), “references the critical insight that race, class, gender, sexuality, ethnicity, nation, ability, and age operate … as reciprocally constructing phenomena that in turn shape complex social inequalities.” (Collins 2015) It is imperative to use any data we have to show how these additional factors influence both the number of injured and the rate of recovery. This information opens a world of possibilities to explore disparities in the occurrence, treatment, and recovery of SRI.

In the US, a person’s opportunity for sport and recreation participation and injury is heavily influenced by a person’s race and/or ethnicity, both social constructs (Jiménez et al. 2015; Yoon et al. 2018; Lambert et al. 2019; Kurka et al. 2015; Harrington et al. 2017; Robinson et al. 2016). Due to long standing blatant and, more recently, neutrally written policies in the US (Sivashanker et al. 2020; Historical Foundations of Race n.d.; Rothstein 2017; Anderson 2018; Hajnal et al. 2017; Powell 2014), Black and Brown people traditionally have less access to care (Flower et al. 2020), opportunity, access to greenspaces (Bruton and Floyd 2014; Engelberg et al. 2016; Browning and Rigolon 2018), and other resources such as well funded schools (Darling-Hammond 2007; Morgan and Amerikaner 2018). They face discrimination in their interactions with law enforcement (Schwartz 2020; Graham et al. 2020; Bor et al. 2018; Edwards et al. 2018; Alang 2018) and the health system (Egede 2006; Alang et al. 2020; Sakran et al. 2020; Institutional Racism in the Health Care System n.d.; Keshavan 2020; Evans et al. 2020; Oribhabor et al. 2020). Generational deficiencies have compounded to form the basis of current health system interactions and outcomes. These communities have higher burdens of health risk factors, worse health outcomes, and reduced access to care (Artiga and Orgera 2019). Just as with COVID-19, even if Black people have insurance and other means of access, they are less likely to get the same level of care as others (Williams 2020; Williams et al. 2003; Odonkor et al. 2020) especially if they report issues of pain (Hoffman et al. 2016; Lee et al. 2019; Aronowitz et al. 2020; Druckman et al. 2018).

Few population level data sources indicate how many people in a population participate in community sport and recreation, or how the injury risk profile might be different between those who participate and those that do not participate for whatever reason. In some situations, data linkage is an ideal way to acquire population level data that applies to the population under concern and prevent duplication of efforts. Many existing SRI data sources rely on athletic trainers to capture all variables. This collection is a severe time burden for collectors – a precious commodity especially for staff working in non-elite sport. With more complete data we could examine variations in injuries and outcomes between settings with athletic trainers and those without, providing more evidence to support expansion of access to athletic trainers. We can use our new data to examine questions other areas of injury have pursued – racial disparities (Hamann et al. 2020), impacts of SES (Goldman et al. 2018) – and take a more advanced statistical approach to questions such as the impact of safe housing, food security, and social networks on initial and subsequent SRI.

In some instances, one could use multi-level modeling to combine population level SDOH independent variables with SRI information. We must also then assume that the population level demographics apply to our study group. We have already stated that Black and Brown athletes make up the majority of participation in organized collegiate sports such as NCAA Division I football and basketball (Lapchick 2020). Because they do not make up the same proportion of all college students and often have different SDOH, it would not be advisable to assume that statistics about all college students apply. We would use publicly available data from a source such as the NCAA to enhance our analysis and provide population specific guidance.

### Challenges

There are three primary roadblocks to incorporating SDOH factors in research – researchers, data creators/managers, and peer-reviewed journals. It is up to the researchers using the information to push for inclusion of SDOH in data that does not contain it, the data creators and managers to make this information’s existence known and available to those that request it, and journals to encourage the inclusion of SDOH in manuscripts submitted for publication. We must be more open to sharing SDOH data and increase the number of collaborations that use it. If data is collected and never used, it becomes less important and costlier to retain in lieu of other possible variables.

We must regularly communicate what we are doing, why we are doing it, and what we find out to those outside of science and medicine. Policy makers and the general public are among the users of the work being conducted about SDOH and about SRI. This means we must make sure to use avenues such as policy briefs, websites, videos, social media, popular magazines, and infographics to share research findings in usable chunks. The policy makers and community can use these findings to achieve their goals and assist in the push for equity.

## Conclusion

Because SDOH are critical to understanding SRI, we must consistently gather information on the who, what, where, when, why, and how related to these determinants. We must know how and where we can make a difference, what intersections we face, and interdisciplinary changes that will help us best develop solutions. We can only achieve equity in SRI by reducing health disparities and we can only identify the disparities if we start with better data collection and utilization. This problem affects everyone and it will take everyone to address it. Together we can make a difference.

## Data Availability

Not applicable.

## Abbreviations

NCAA: The National Collegiate Athletics Association
SDOH: Social Determinants of Health
SES: Socioeconomic Status
SRI: Sport and Recreation Injury(ies)
